# Microbial Quotients and Stoichiometric Imbalance Reflect Divergent Responses to Wetland Land-Use Conversion

**DOI:** 10.3390/microorganisms14081844

**Published:** 2026-08-19

**Authors:** Zehao Wang, Ye Li, Jiuwang Jin

**Affiliations:** Key Lab of Eco-Restoration of Regional Contaminated Environment, Ministry of Education, Shenyang University, Shenyang 110044, China; wangzh021202@163.com (Z.W.); m18341014857@163.com (J.J.)

**Keywords:** wetland degradation, land-use conversion, microbial quotient, stoichiometric imbalance, microbial biomass

## Abstract

Wetland degradation alters soil nutrient cycling and microbial functioning, yet how microorganisms adjust resource-use strategies after wetland conversion remains poorly understood. This study examined the effects of wetland degradation-induced land-use conversion on soil-microbial quotients (qMBC, qMBN, qMBP) and soil-microbial stoichiometric imbalance (C:Nimb, C:Pimb, N:Pimb) in the A’er Xiang lake marsh wetland, northeastern China. Five sites were compared: native wetland, 5- and 10-year forestland converted from wetland, and 5- and 10-year cropland converted from wetland. The qMBP increased from 11.15% in native wetland to 30.40% in 10-year cropland, while qMBC rose from 5.97% to 9.81%, indicating that microbial P and C quotients were highly sensitive to wetland conversion. C:Nimb increased to 2.93 in 10-year cropland, whereas N:Pimb rose from 4.93 in native wetland to 13.79 and 10.59 in 5-year and 10-year forestland, respectively. Correlation analysis demonstrated that soil, microbial, and enzyme stoichiometry jointly regulated microbial quotients. Specifically, C:Nimb was strongly and negatively correlated with N:Pimb (r = −0.839, *p* < 0.01), and microbial biomass showed contrasting responses, being positively correlated with C:Nimb but negatively correlated with C:Pimb and N:Pimb. These indicators can guide future land-use decisions by integrating water availability with microbial adaptation in degraded sandy wetland regions.

## 1. Introduction

Wetlands are key ecosystems for maintaining biodiversity, regulating hydrological processes, storing carbon and supporting regional ecological stability [1]. However, climate change, agricultural expansion and other human disturbances have accelerated wetland degradation and land-use conversion in many regions [2,3]. Wetland degradation and subsequent conversion to cropland or forestland fundamentally alter plant community composition, as native wetland vegetation is replaced by agricultural crops such as maize on cropland, or by trees and shrubs on forestland. This shift in vegetation type drives changes in soil physicochemical properties and in the taxonomic composition and diversity of soil-microbial communities, including both alpha- and beta-diversity [2]. The underlying mechanism is that plant species differ markedly in litter quality, root exudate profiles, and nutrient uptake strategies, which in turn regulate soil organic matter inputs, pH, moisture regimes, and the resources available to soil microbes [3]. Since soil microorganisms are the primary agents of organic matter decomposition, nitrogen transformation, and phosphorus mobilization, any alteration in their community composition and diversity can directly reshape the rates and pathways of nutrient cycling, ultimately influencing overall ecosystem functioning.

Soil microorganisms play central roles in organic matter decomposition, nutrient transformation and biogeochemical cycling [4]. Because microbial biomass represents an active nutrient pool, changes in microbial biomass C, N and P can provide early evidence of altered soil ecological function under land-use change [5,6]. Previous work in the A’er Xiang wetland showed that wetland degradation and conversion to forestland or cropland significantly altered soil, microbial and extracellular enzyme C, N and P contents and their stoichiometric characteristics [7]. However, changes in nutrient pools alone do not fully explain whether microbial resource demand remains matched with soil nutrient supply during land-use conversion.

Ecological stoichiometry reveals the principles governing elemental exchange between organisms and their environment, with its central focus on the shifts in elemental composition and stoichiometric structure driven by biological activity. It provides a useful framework for linking soil nutrient supply, microbial elemental composition and extracellular enzyme allocation [8,9]. Soil C:N:P stoichiometry is sensitive to climate, vegetation, soil properties and land-use change, especially in ecologically fragile regions [8,10]. Land-use conversion can alter the C:N:P stoichiometry of soils, microorganisms and enzymes, and these changes may become more pronounced with increasing conversion duration [9,11]. Enzyme stoichiometry can further indicate microbial nutrient-acquisition strategies because C-, N- and P-acquiring enzymes reflect microbial investment in different resource pools [12]. Because stoichiometric balances integrate the coupled dynamics of nutrient inputs from vegetation, nutrient storage in soil, and nutrient turnover driven by microbial metabolism, a change in plant community composition and microbial diversity is expected to manifest as a shift in soil C:N:P stoichiometry. This shift can serve as a sensitive indicator of nutrient limitation, cycling efficiency, and ecosystem stability under wetland degradation and land-use conversion scenarios.

Microbial quotients, commonly expressed as the ratios of microbial biomass C, N, and P to the corresponding soil organic C, total N, and total P pools, are widely used to indicate the relative contribution of microbial biomass to soil nutrient pools and to assess microbial substrate use efficiency [13,14]. Compared with microbial biomass alone, qMBC (quotient of microbial biomass C), qMBN (quotient of microbial biomass N) and qMBP (quotient of microbial biomass P) can provide additional information on microbial resource-use efficiency and the active fraction of soil nutrients [13,14]. Studies in grassland and cropland ecosystems have shown that microbial quotients respond sensitively to land-use patterns, soil erosion and soil physicochemical changes [5,14]. Therefore, microbial quotients may be useful for identifying microbial functional shifts during wetland degradation-induced land-use conversion.

Soil-microbial stoichiometric imbalance is another important indicator of microbial adaptation to changing resource conditions [15,16]. Stoichiometric imbalance occurs when soil resource stoichiometry deviates from microbial biomass stoichiometry, potentially leading to microbial nutrient limitation and altered C, N and P cycling [15]. Recent studies have demonstrated that stoichiometric imbalance can constrain microbial C and N dynamics and influence microbial nutrient-use efficiency [15,16]. However, the responses of microbial quotients and soil-microbial stoichiometric imbalance to wetland conversion remain less explicit than changes in soil nutrient pools or enzyme activities.

The A’er Xiang wetland is a typical inland lake marsh wetland located along the southern margin of the Horqin Sandy Land, where a sandy land-lake marsh wetland complex has undergone substantial degradation [7]. This land-use mosaic provides a suitable field setting for comparing microbial resource-use responses under different wetland conversion pathways. Agricultural conversion can homogenize soil-microbial communities and alter microbial C cycling, while land-use intensification may also reduce microbial C use efficiency through changes in soil stoichiometry [17,18,19].

In this study, we focused on soil-microbial quotients and soil-microbial stoichiometric imbalance rather than repeating the previously reported soil–microbe–enzyme nutrient pool perspective [7]. Specifically, we aimed to: (1) determine how wetland conversion to forestland and cropland affects qMBC, qMBN and qMBP; (2) evaluate the responses of C:Nimb, C:Pimb and N:Pimb under different conversion pathways; and (3) clarify the relationships among microbial biomass, microbial quotients, soil-microbial stoichiometric imbalance and extracellular enzyme stoichiometry. We hypothesized that forestland and cropland conversion would produce divergent microbial resource-use patterns. We further hypothesized that cropland conversion would increase microbial quotients but intensify C:N imbalance, whereas forestland conversion would mainly enhance N:P imbalance.

## 2. Materials and Methods

### 2.1. Study Area

**Figure 1:**
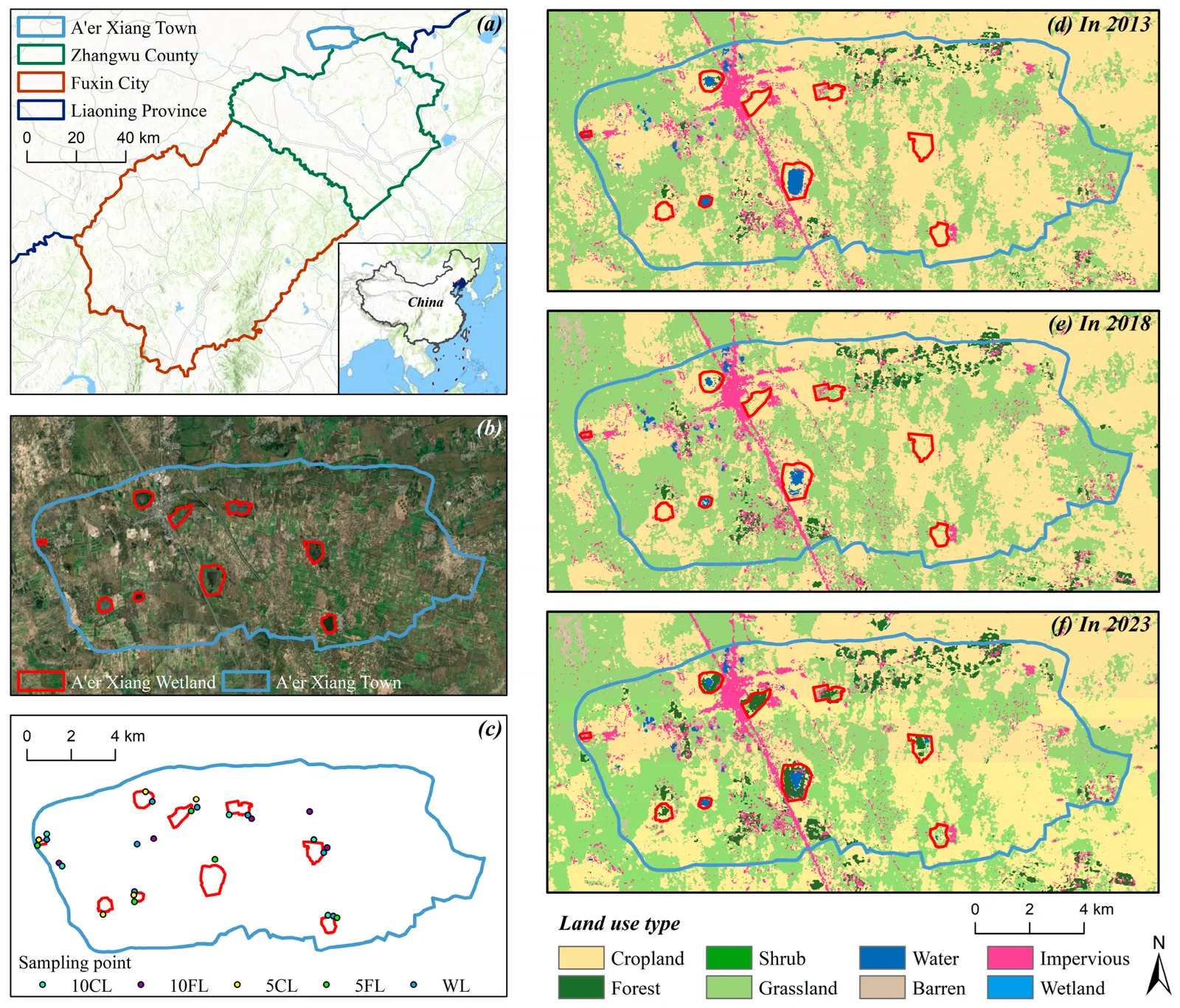
Study area location, sampling sites, and temporal land-use type transitions in A’er Xiang wetland. (**a**) Location of the study region in Liaoning Province, Northeast China; (**b**) identification of the A’er Xiang wetland area; (**c**) spatial arrangement of the sampling sites; (**d**–**f**) spatial distribution of various land-use types in the years 2013, 2018, and 2023.

The study was conducted in the A’er Xiang wetland, located in the northern part of Zhangwu County, Fuxin City, Liaoning Province, China. The study area extends from 122°20′02″ to 122°34′01″ E and from 42°43′15″ to 42°50′05″ N, and lies along the southern margin of the Horqin Sandy Land (Figure 1). This region is situated in an ecologically fragile transition zone between arid and humid environments in northeast Asia and also represents an agro-pastoral ecotone in western Liaoning. The total area of the study region is approximately 137 km^2^, with an average elevation of 220–260 m [7].

The A’er Xiang wetland is characterized by a distinctive sandy land–lake marsh wetland complex, where mobile, semi-mobile, and fixed dunes, wind-eroded depressions, and interdune lowlands are widely distributed. The interaction between aeolian sand movement and wind-eroded depressions has shaped a typical dune–marsh landscape, with sand deposition and blocking processes influencing the hydrological supply of lake marsh wetlands. This landscape is underlain by thick quaternary fluvial and aeolian sand deposits reaching 100–200 m in depth, and no bedrock outcrops occur. The deeply buried bedrock exerts negligible influence on surface topography, so the present dune–interdune relief is entirely shaped by aeolian processes, resulting in a heterogeneous landscape characterized by alternating elliptical dune clusters and wind-eroded depressions. The soils developed from these ancient river alluvial sandy parent materials are predominantly meadow aeolian sandy soil and mobile aeolian sandy soil, which correspond to Aerosols in the World Reference Base for Soil Resources. Owing to weak soil-forming processes, the profiles are loose, structureless, and generally nutrient-poor, with very low organic matter content. Morphologically, the surface horizon is typically gray to brownish, the subsurface horizon is yellowish and occasionally stained brownish-yellow by iron oxides, and the bottom layer consists of yellowish-white sand, with neutral pH throughout the profile. In the waterlogged wetland landscape, these sandy soils perform several essential ecological functions, including regulating water infiltration, filtering surface runoff, and providing substrates for anaerobic microbial processes such as denitrification and organic matter decomposition. During wetland degradation, the hydrological regime of these soils follows a progressive desiccation sequence, from perennial inundation through seasonal water saturation to semi-arid moisture conditions, driven by declining groundwater tables. This drying trend leads to gradual shifts in soil redox status, a decline in organic matter accumulation, and increased mineralization rates, fundamentally altering the soils’ capacity for nutrient retention and hydrological buffering.

The region has a temperate continental monsoon climate with semi-arid and wind-sandy characteristics. The annual mean temperature ranges from 5.7 to 7.6 °C, and the annual mean precipitation is approximately 550 mm, more than 66% of which occurs from June to August. Annual evaporation ranges from 1300 to 1800 mm. Surface runoff are limited because of the high infiltration capacity and weak water-holding capacity of sandy soil, whereas groundwater is relatively abundant and mainly occurs as alluvial phreatic water at a depth of approximately 3–5 m.

Since 1985, the A’er Xiang wetland has undergone severe degradation. Monitoring data showed that the wetland area decreased from 8.32 km^2^ in 1985 to 0.91 km^2^ in 2019, corresponding to a degradation rate of approximately 89%. After degradation, part of the original wetland was converted to forestland to control desertification and protect water sources, whereas another part was converted to cropland because of agricultural expansion and increasing demand for land and water resources (Figure 1). Before 2013, management of these converted areas was not yet organized into a stable regional program; systematic afforestation and agricultural management began in 2013, when the local government launched a campaign to combat desertification along the southern margin of the Horqin Sandy Land. Since then, forestland has been planted almost exclusively with *Pinus sylvestris* var. *mongolica* and *Pinus tabuliformis*, and the tree species composition has remained consistent across all forestland sites. Cropland has been continuously cultivated with maize under conventional tillage and standard local fertilization regimes, with no major changes in cropping practices over the conversion period. As a result, we took the year 2013 as the baseline for wetland conversion, and designated 2018 and 2023 as the two evaluation time points corresponding to five-year and ten-year conversion durations. Sampling was conducted in 2023, allowing a space-for-time assessment of the current status of the five treatment groups. By 2023, the land-use pattern in the A’er Xiang area had become relatively stable, and this setting therefore provides a suitable field system for examining how wetland degradation-induced land-use conversion affects soil-microbial quotients and soil-microbial stoichiometric imbalance.

### 2.2. Soil Sampling and Laboratory Analysis

Based on satellite images of the study area over the past decade, degraded wetland areas were identified, and the areas converted from wetland to forestland and cropland were delineated. A space-for-time substitution approach was used to select areas with different conversion durations. Five treatment groups were established: native wetland (WL), wetland converted to forestland for five years (5FL), wetland converted to forestland for ten years (10FL), wetland converted to cropland for five years (5CL), and wetland converted to cropland for ten years (10CL) (Figure 1c) (Table 1) [7]. For each land-use type, three independent replicate plots were established, yielding a total of 15 plots.

Soil samples were collected at the end of October 2023, which coincides with the autumn harvest and plant senescence period in northern China. During this season, the soil is markedly affected by both natural environmental factors and anthropogenic activities, leading to pronounced differences among the five treatment groups. This makes the autumn period particularly suitable for exploring the response mechanisms of soil endogenous substances under land-use conversion. Moreover, soil-microbial populations increase substantially in autumn, and their metabolic activity becomes more vigorous. Therefore, soil collected at this time point is more representative for the purposes of this study.

In each plot, a 1 m^2^ sampling area was delimited, and five soil cores were collected from the 0–20 cm layer using a quincunx sampling design. The 0–20 cm surface layer was selected as the core sampling horizon because it is the zone most directly affected by anthropogenic activities, land-use conversion, and wetland degradation, and therefore best reflects the immediate impacts on soil properties. Plant roots and litter were removed in the field. A portion of each soil sample was placed in aluminum boxes and immediately transported to the laboratory for soil moisture determination. The remaining soil was sealed in self-sealing bags and transported to the laboratory. One portion was air-dried naturally in a ventilated place, ground, passed through 20-mesh and 100-mesh sieves, and stored for the determination of soil organic carbon (SOC), total nitrogen (TN) and total phosphorus (TP). Another portion was stored at 4 °C for the determination of microbial biomass and extracellular enzyme activity. Prior to analysis, soil moisture content was adjusted according to the requirements of the national standard methods.

SOC was determined using the potassium dichromate oxidation method, TN was measured using the Kjeldahl method, and TP was determined using the molybdenum–antimony colorimetric method after alkaline fusion. Microbial biomass carbon (MBC) was determined using the chloroform fumigation-K_2_SO_4_ extraction method [20]. Microbial biomass nitrogen (MBN) was determined using the chloroform fumigation-K_2_SO_4_ extraction method [21]. Microbial biomass phosphorus (MBP) was determined using the chloroform fumigation-NaHCO_3_ extraction-colorimetric method [22]. These fumigation-extraction methods are widely used to quantify active microbial C, N and P pools in soils.

The activities of extracellular enzymes involved in C, N and P acquisition were also measured. β-1,4-glucosidase (BG) was used as the C-acquiring enzyme, β-1,4-N-acetylglucosaminidase (NAG) and L-leucine aminopeptidase (LAP) were used as N-acquiring enzymes, and phosphatase (PHOS) was used as the P-acquiring enzyme. Enzyme activities were determined using the microplate fluorescence method, which is commonly applied to assess potential hydrolytic enzyme activities in soil ecological studies [21]. Fresh soil was mixed with sodium acetate buffer, shaken, and then loaded into 96-well microplates with corresponding enzyme substrates. After incubation in the dark, the reaction was terminated with NaOH, and fluorescence was measured using a multifunctional microplate reader at an excitation wavelength of 365 nm and an emission wavelength of 450 nm. All measurements were conducted with three parallel replicates.

### 2.3. Calculation and Statistical Analysis

Microbial biomass C, N and P were calculated from the differences between fumigated and non-fumigated soil extracts [20,21,22]. MBC and MBN were calculated as:

(1) *MBC* = *E_C_*/*k_EC_*

(2) *MBN* = *E_N_*/*k_EN_*

where *E_C_* and *E_N_* are the differences in extracted organic carbon and nitrogen between fumigated and non-fumigated soils, respectively. The conversion coefficients *k_EC_* and *k_EN_* were both 0.45 [20,21].

MBP was calculated as:

(3) *MBP* = *E_Pi_*/(*k_P_* × *R_Pi_*)

where *E_Pi_* is the difference in extracted phosphorus between fumigated and non-fumigated soils, *R_Pi_* is the phosphorus recovery rate, and *k_P_* is the conversion coefficient, with a value of 0.4 [22].

Extracellular enzyme activity (EEA) stoichiometric ratios were calculated as [12,21]:

(4) *EEA_(C:N)* = *BG*/(*NAG + LAP*)

(5) *EEA_(C:P)* = *BG*/*PHOS*

(6) *EEA_(N:P)* = (*NAG* + *LAP*)/*PHOS*

where *BG* represents β-1,4-glucosidase activity, *NAG + LAP* represents the combined activity of N-acquiring enzymes, and *PHOS* represents phosphatase activity.

Soil-microbial quotients were calculated as [13,14]:

(7) *qMBC* = *MBC*/*SOC*

(8) *qMBN* = *MBN*/*TN*

(9) *qMBP* = *MBP*/*TP*

where *qMBC*, *qMBN* and *qMBP* represent microbial *C*, *N* and *P* quotients, respectively.

Soil-microbial stoichiometric imbalance was calculated as [15,16]:

(10) *C:N_imb_* = (*SOC*/*TN*)/(*MBC*/*MBN*)

(11) *C:P_imb_* = (*SOC*/*TP*)/(*MBC*/*MBP*)

(12) *N:P_imb_* = (*TN*/*TP*)/(*MBN*/*MBP*)

where *C:N_imb_*, *C:P_imb_* and *N:P_imb_* represent soil-microbial *C:N*, *C:P* and *N:P* stoichiometric imbalance, respectively.

All raw data were organized using Excel 2019. Soil C, N and P data were compared with Fuxin soil data from the Second National Soil Survey of China to determine the allowable error range. Potential outliers were identified using boxplots and were corrected by mean replacement or removed when necessary. Statistical analyses were conducted using SPSS version 19.0. Normality and homogeneity of variance were tested before significance analysis. One-way analysis of variance (ANOVA) was used to test differences among land-use/conversion types, and the least significant difference (LSD) method was used for multiple comparisons. Pearson correlation analysis was used to examine the relationships among microbial biomass, soil-microbial quotients, soil-microbial stoichiometric imbalance, soil stoichiometry and extracellular enzyme stoichiometry. Figures were generated using Arcgis 10.8 and Origin 2021. Different lowercase letters indicate significant differences among groups at *p* < 0.05, and significance levels were defined as * *p* < 0.05, ** *p* < 0.01, and *** *p* < 0.001. To minimize systematic and experimental errors, blank controls and three parallel replicates were included in the experimental process, and all data are presented as mean ± standard deviation.

## 3. Results

### 3.1. Effects of Land-Use Conversion on Soil-Microbial Quotients

**Figure 2:**
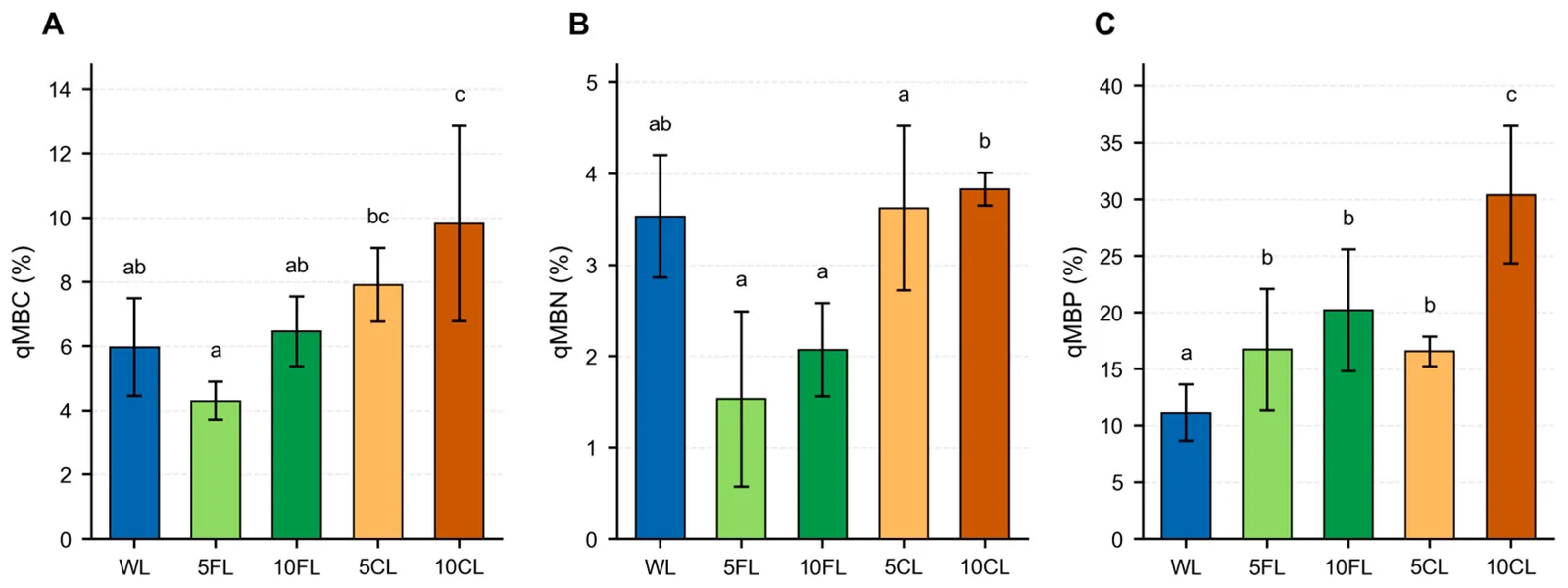
Changes in soil-microbial quotients among the five treatment groups. (**A**) qMBC; (**B**) qMBN; (**C**) qMBP. Bars represent means, and error bars represent standard deviations. Different lowercase letters indicate significant differences among the five treatment groups at *p* < 0.05.

Soil-microbial quotients varied markedly among the five treatment groups (Figure 2). The qMBC values ranged from 4.29 to 9.81%, with the lowest value observed in 5FL and the highest value observed in 10CL. Compared with WL, qMBC decreased after five years of forestland conversion, but increased again in 10FL. In cropland conversion sites, qMBC showed a continuous increasing trend with conversion duration, increasing from 7.91% in 5CL to 9.81% in 10CL. The qMBC value in 10CL was significantly higher than that in WL, 5FL and 10FL, whereas no significant difference was observed between 5CL and 10CL (Figure 2A).

The response pattern of qMBN differed from that of qMBC. The qMBN value was 3.53% in WL, decreased to 1.53% in 5FL and 2.07% in 10FL, and increased to 3.62% and 3.83% in 5CL and 10CL, respectively. Among the five treatment groups, 10CL showed the highest qMBN value, whereas 5FL showed the lowest value. The qMBN value in 10CL was significantly higher than those in 5FL, 10FL and 5CL, but it was not significantly different from that in WL (Figure 2B).

The qMBP value showed a stronger response to wetland degradation-induced land-use conversion than qMBC and qMBN. The qMBP value was lowest in WL, with a value of 11.15%, and increased significantly after conversion to forestland and cropland. The qMBP values in 5FL, 10FL and 5CL were 16.74%, 20.19% and 16.57%, respectively, and were all significantly higher than that in WL. The highest qMBP value occurred in 10CL, reaching 30.40%, which was significantly higher than those in the other four treatment groups (Figure 2C). Overall, these results indicate that microbial P quotients were the most sensitive microbial quotients index in response to wetland degradation-induced land-use conversion.

### 3.2. Effects of Land-Use Conversion on Soil-Microbial Stoichiometric Imbalance

**Figure 3:**
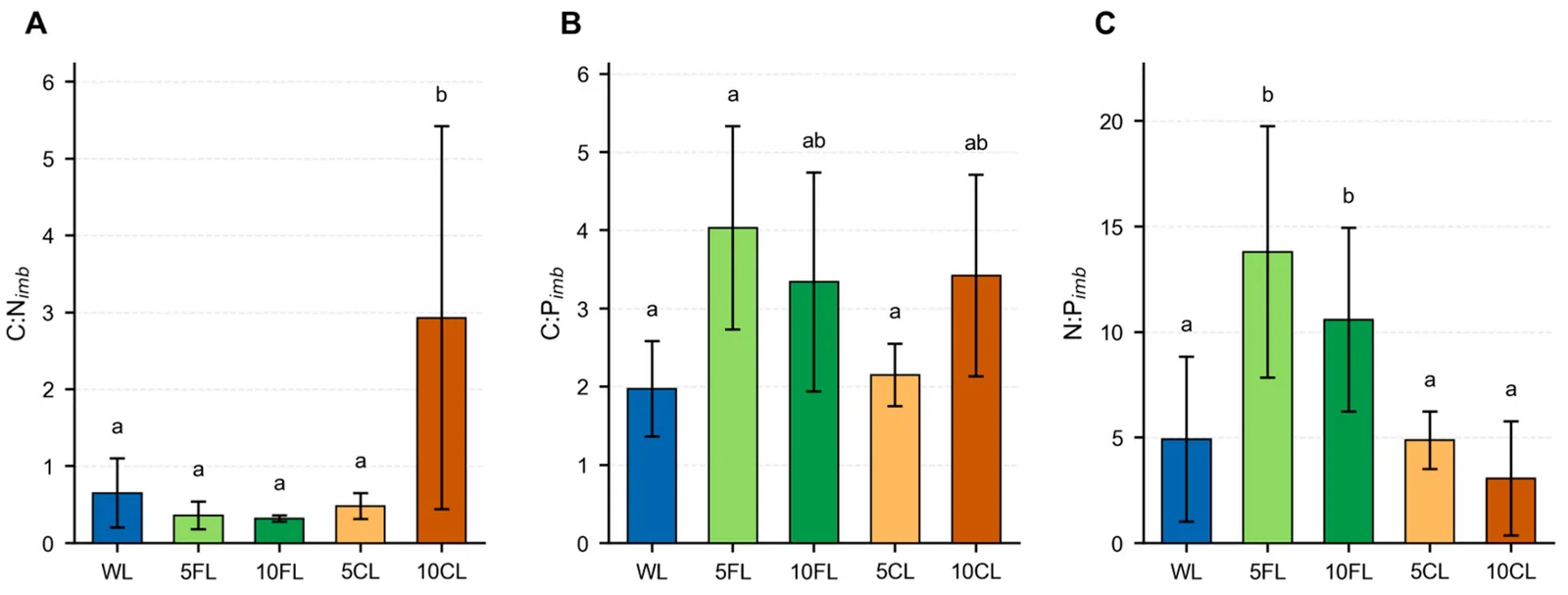
Changes in soil-microbial stoichiometric imbalance among the five treatment groups. (**A**) C:Nimb; (**B**) C:Pimb; (**C**) N:Pimb. Bars represent means, and error bars represent standard deviations. Different lowercase letters indicate significant differences among the five treatment groups at *p* < 0.05.

Soil-microbial stoichiometric imbalance also differed among the five treatment groups (Figure 3). The C:Nimb values were relatively low in WL, 5FL, 10FL and 5CL, ranging from 0.32 to 0.65, with no significant differences among these four types. However, C:Nimb increased sharply in 10CL, reaching 2.93, which was significantly higher than those in all other treatment groups (Figure 3A). This result indicates that long-term cropland conversion strongly enhanced the imbalance between soil C:N supply and microbial C:N demand.

The C:Pimb values ranged from 1.97 to 4.03 across the five treatment groups. The highest mean value occurred in 5FL, followed by 10CL and 10FL, whereas WL and 5CL showed relatively lower values. However, because of the large variation within groups, C:Pimb did not show significant differences among the five treatment groups (Figure 3B). This suggests that the response of soil-microbial C:P imbalance to wetland conversion was less consistent than that of C:Nimb and N:Pimb.

The N:Pimb values showed a distinct pattern between forestland and cropland conversion pathways. Compared with WL, N:Pimb increased significantly in both 5FL and 10FL, with values of 13.79 and 10.59, respectively. In contrast, N:Pimb remained relatively low in cropland conversion sites, with values of 4.87 in 5CL and 3.06 in 10CL, neither of which differed significantly from WL (Figure 3C). These results suggest that forestland conversion mainly enhanced soil-microbial N:P imbalance, whereas cropland conversion did not significantly increase N:Pimb.

### 3.3. Relationships Between Microbial Biomass and Soil-Microbial Stoichiometric Imbalance

**Figure 4:**
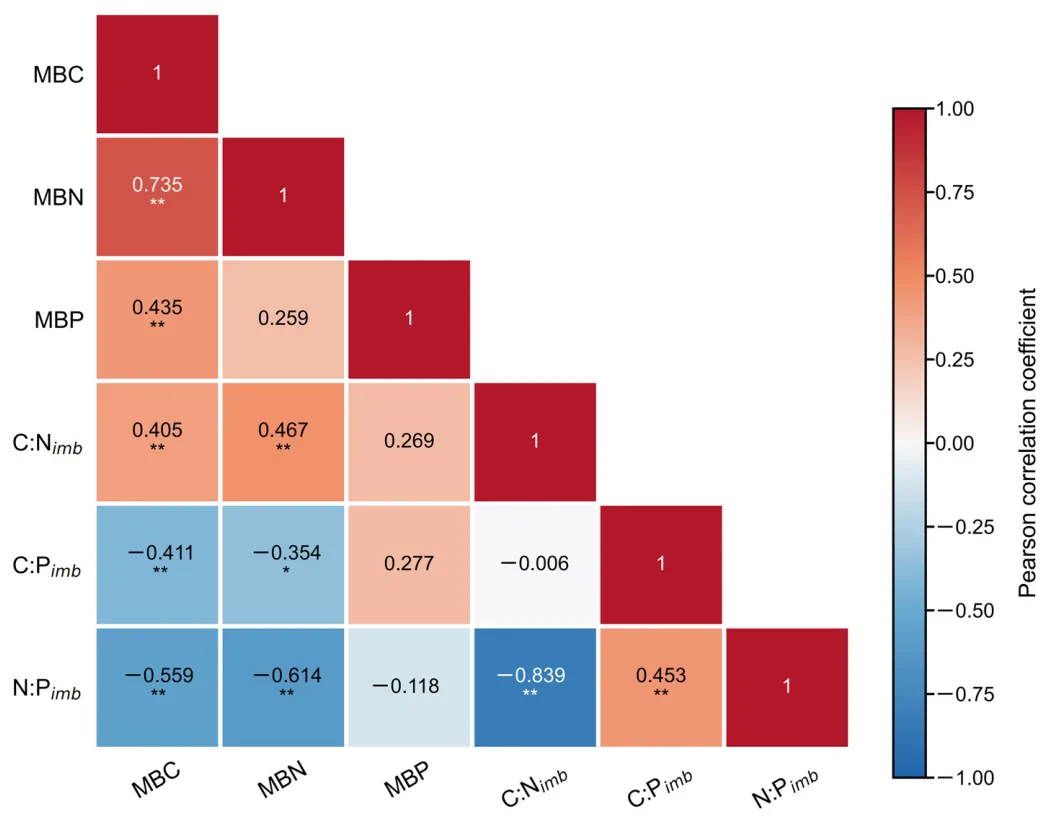
Pearson correlations between microbial biomass and soil-microbial stoichiometric imbalance. Red indicates positive correlations, and blue indicates negative correlations. Color intensity represents the magnitude of the correlation coefficient. * and ** indicate significant correlations at *p* < 0.05 and *p* < 0.01, respectively.

Pearson correlation analysis showed clear relationships between microbial biomass and soil-microbial stoichiometric imbalance (Figure 4). MBC was significantly and positively correlated with MBN and MBP, with correlation coefficients of 0.735 and 0.435, respectively. MBC was also significantly positively correlated with C:Nimb, but significantly negatively correlated with C:Pimb and N:Pimb. These results indicate that microbial biomass C accumulation was closely associated with changes in soil-microbial stoichiometric imbalance, especially the decrease in C:Pimb and N:Pimb.

MBN showed a similar correlation pattern. It was significantly positively correlated with MBC and C:Nimb, but significantly negatively correlated with C:Pimb and N:Pimb. In contrast, MBP showed a significant positive correlation only with MBC, while its correlations with C:Nimb, C:Pimb and N:Pimb were not significant (Figure 4). This suggests that microbial C and N were more closely linked to soil-microbial stoichiometric imbalance than microbial P.

Among the imbalance indices, C:Nimb was significantly and negatively correlated with N:Pimb, with a correlation coefficient of −0.839, whereas C:Pimb was significantly and positively correlated with N:Pimb, with a correlation coefficient of 0.453. No significant relationship was observed between C:Nimb and C:Pimb (Figure 4). These results indicate that the different dimensions of soil-microbial stoichiometric imbalance were not independent, and that changes in N:Pimb were closely associated with both C:Nimb and C:Pimb.

### 3.4. Correlation Analysis Among Soil Stoichiometry, Microbial Quotients, and Extracellular Enzyme Stoichiometry

**Figure 5:**
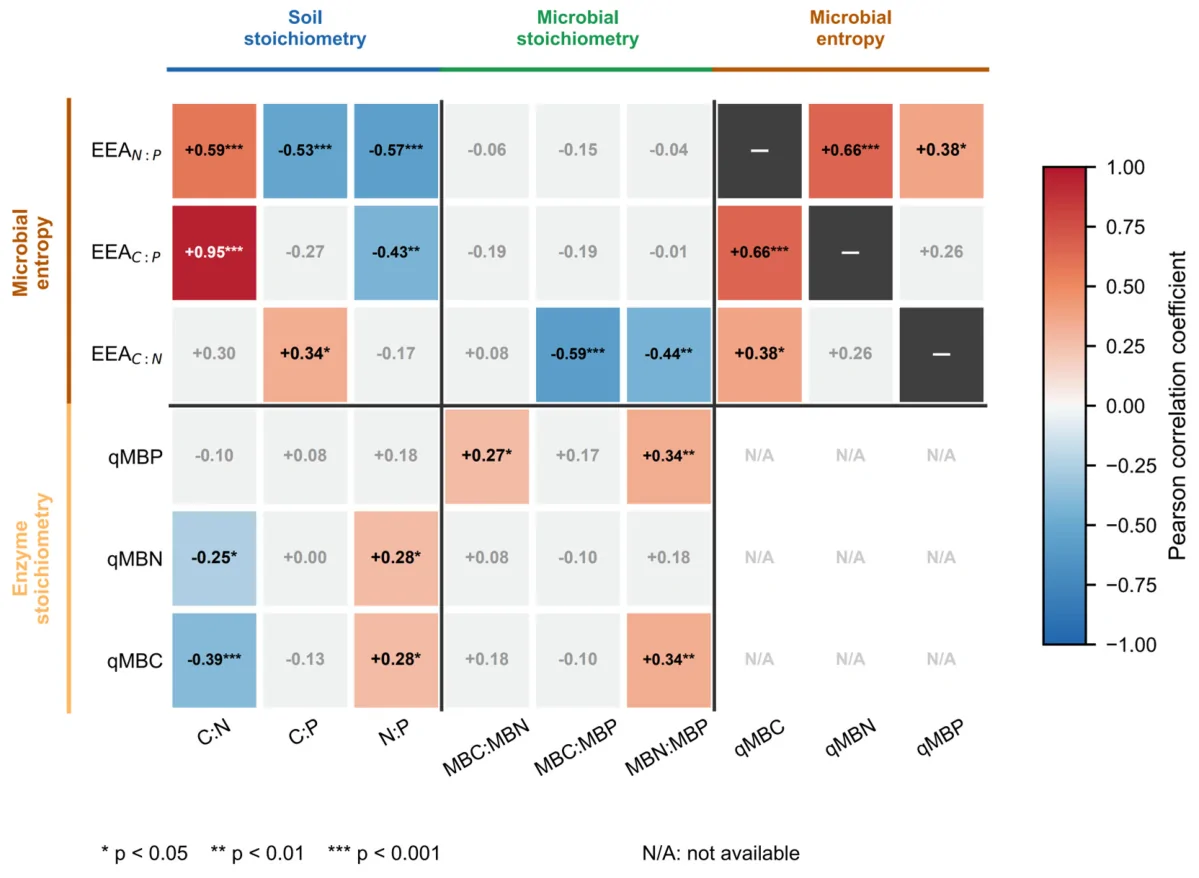
Pearson correlations among soil stoichiometry, microbial stoichiometry, microbial quotients, and extracellular enzyme stoichiometry. Red indicates positive correlations, and blue indicates negative correlations. Color intensity represents the magnitude of the correlation coefficient. *, **, and *** indicate significant correlations at *p* < 0.05, *p* < 0.01, and *p* < 0.001, respectively.

Pearson correlation analysis revealed the correlation analysis among soil stoichiometry, microbial stoichiometry, microbial quotients, and extracellular enzyme stoichiometry (Figure 5). Soil stoichiometric ratios showed significant correlations with both extracellular enzyme stoichiometry and microbial quotients. Soil C:N was strongly and positively correlated with EEA C:P (r = 0.95, *p* < 0.001) and EEA N:P (r = 0.59, *p* < 0.001), but was negatively correlated with qMBC (r = −0.39, *p* < 0.001) and qMBN (r = −0.25, *p* < 0.05). In contrast, soil N:P was negatively correlated with EEA N:P (r = −0.57, *p* < 0.001) and EEA C:P (r = −0.43, *p* < 0.05), but was positively correlated with qMBC (r = 0.28, *p* < 0.05) and qMBN (r = 0.28, *p* < 0.05). These results indicate that soil C:N and N:P ratios linked enzyme stoichiometry and microbial quotients in opposite directions.

Microbial stoichiometric ratios showed selective correlations with enzyme stoichiometry and microbial quotients. EEA C:N was negatively correlated with MBC:MBP (r = −0.59, *p* < 0.001) and MBN:MBP (r = −0.44, *p* < 0.01), suggesting that microbial biomass P stoichiometry was closely associated with enzyme C:N allocation. Among the microbial quotient indices, qMBP was positively correlated with MBC:MBN (r = 0.27, *p* < 0.05) and MBN:MBP (r = 0.34, *p* < 0.01), while qMBC was positively correlated with MBN:MBP (r = 0.34, *p* < 0.01). These relationships indicate that microbial biomass P stoichiometry served as a link between enzyme stoichiometry and microbial quotients.

Extracellular enzyme stoichiometry was directly correlated with microbial quotients. EEA C:P was positively correlated with qMBC (r = 0.66, *p* < 0.001), and EEA N:P was positively correlated with qMBN (r = 0.66, *p* < 0.001) and qMBP (r = 0.38, *p* < 0.05). EEA C:N was also positively correlated with qMBC (r = 0.38, *p* < 0.05). These results indicate that enzyme C:P and N:P stoichiometry were closely linked to microbial C and N quotients, respectively. In contrast, qMBP showed no significant correlations with any soil stoichiometric ratio, suggesting that the microbial P quotient may be regulated more by microbial biomass stoichiometry than by soil nutrient ratios.

Overall, the correlation analysis indicates that soil stoichiometry, particularly C:N and N:P ratios, served as key links between extracellular enzyme stoichiometry and microbial quotients. Microbial biomass stoichiometry, especially P-related ratios, provided additional connections between enzyme stoichiometry and microbial quotients. These correlation analyses suggest that soil nutrient supply, microbial biomass stoichiometry, and enzyme-mediated nutrient acquisition jointly regulate microbial quotients in this wetland degradation system.

## 4. Discussion

### 4.1. Soil-Microbial Quotients as a Sensitive Indicator of Wetland Degradation

Soil-microbial quotients reflect the proportion of microbial biomass C, N and P in the corresponding soil nutrient pools, and therefore provide information beyond microbial biomass alone [13,14]. Compared with microbial biomass content, microbial quotients can better indicate the relative contribution of the microbial pool to soil nutrient cycling and the active fraction of soil nutrients [13,14]. In this study, qMBC, qMBN and qMBP showed distinct responses to wetland degradation-induced land-use conversion (Figure 2). The qMBC and qMBP values were highest in 10CL, and qMBN was also relatively high in cropland conversion sites. These results suggest that the long-term conversion of wetland to cropland increased the relative contribution of microbial biomass to soil C, N and P pools, especially for microbial C and P quotients.

The response of qMBP was particularly strong. Compared with WL, all converted sites showed significantly higher qMBP, and 10CL showed the highest value among the five treatment groups (Figure 2C). This indicates that the microbial P quotient may be more sensitive than microbial C and N quotients to wetland degradation and subsequent land-use conversion. Global synthesis has shown that microbial quotients of C, N and P can differ strongly among ecosystems, suggesting that qMBC, qMBN and qMBP may respond differently to environmental constraints [13]. The strong increase in qMBP in this study may indicate enhanced microbial P immobilization or turnover after wetland conversion, especially under cropland use.

The decrease in qMBC and qMBN in 5FL suggests that short-term conversion to forestland may temporarily reduce the proportion of microbial C and N in soil C and N pools. Similar studies have shown that microbial biomass and microbial quotients can respond sensitively to soil properties, erosion intensity and land-use patterns [5,14]. However, qMBC and qMBP increased in 10FL compared with 5FL, indicating gradual microbial adjustment with increasing conversion duration. This pattern suggests that microbial quotients can sensitively capture the transitional state of soil-microbial functioning during wetland degradation and land-use conversion.

### 4.2. Divergent Responses of Microbial Quotients Under Forestland and Cropland Conversion

Forestland and cropland conversion showed different microbial quotient response pathways. In forestland, qMBC and qMBN were lowest in 5FL, but both increased in 10FL, while qMBP remained significantly higher than that in WL (Figure 2). This pattern indicates that microbial C and N accumulation may have been constrained during the early stage of forestland conversion. Such constraints may be related to the sandy soil background, weak soil development and limited nutrient availability in the A’er Xiang region.

In contrast, cropland conversion was characterized by higher qMBC, qMBN and qMBP, especially in 10CL. This suggests that cropland conversion increased the active microbial nutrient fraction relative to soil nutrient pools. Crop growth, root input, fertilization and agricultural management may provide more available substrates for microorganisms, thereby promoting microbial biomass formation [6,17]. However, this increase should not be interpreted simply as ecological recovery. Agricultural conversion can homogenize soil-microbial communities, and intensive land use can alter microbial C-cycling potential and accelerate nutrient turnover [17,18].

The contrasting responses between forestland and cropland suggest that microbial quotients are strongly pathway dependent. Different land-use types in wetland regions can show distinct microbial community composition and soil properties, and restoration chronosequences can also cause clear changes in microbial community structure [23,24]. In the present study, forestland conversion may represent a gradual adjustment or partial recovery process, whereas cropland conversion may represent a disturbance-driven high-turnover process. Therefore, changes in microbial quotients should be interpreted together with stoichiometric imbalance to distinguish between microbial recovery and disturbance-enhanced microbial activity.

### 4.3. Stoichiometric Imbalance Reveals Resource Mismatch Between Soil Supply and Microbial Demand

Soil-microbial stoichiometric imbalance provides a useful perspective for evaluating the mismatch between soil nutrient supply and microbial elemental demand [15,16]. Stoichiometric imbalance can constrain microbial-driven C and N dynamics because microorganisms must regulate growth, nutrient immobilization and resource allocation when soil resource ratios deviate from microbial biomass stoichiometry [15]. In this study, C:Nimb increased significantly only in 10CL, while WL, 5FL, 10FL and 5CL showed relatively low values (Figure 3A). This indicates that long-term cropland conversion intensified the mismatch between soil C:N supply and microbial C:N demand. The high variability of C:Nimb in 10CL also suggests that cropland conversion may lead to unstable microbial C and N resource matching.

The response of N:Pimb differed from that of C:Nimb. N:Pimb was significantly higher in 5FL and 10FL than in WL, but remained relatively low in cropland conversion sites (Figure 3C). This suggests that forestland conversion mainly enhanced soil-microbial N:P imbalance. Previous work on forest conversion has demonstrated that soil-microbial communities can adjust to stoichiometric imbalances through shifts in community composition, nutrient acquisition, and resource allocation [16]. Such a pattern may also be related to changes in vegetation type, litter input, microbial P accumulation and the nutrient-poor sandy soil background of the study area.

C:Pimb showed no significant differences among the five treatment groups, although relatively high values were observed in 5FL, 10FL and 10CL (Figure 3B). This result indicates that soil-microbial C:P imbalance was more variable and less directly responsive to land-use conversion than C:Nimb and N:Pimb. Previous wetland studies have shown that microbial P limitation and P-cycling functions can vary strongly with flooding regime, wetland type and degradation stage [12,25,26]. Wetland degradation can also alter bacterial and fungal community structure and function, which may further influence microbial nutrient acquisition and stoichiometric adjustment [25,26]. Wetland types and soil properties can shape microbial communities, and long-term degradation can shift microbial functional diversity associated with soil P cycling [27,28]. Therefore, C:Nimb and N:Pimb may be more useful indicators for distinguishing the effects of cropland and forestland conversion in this wetland degradation system.

The correlation analysis further supports the role of stoichiometric imbalance in regulating microbial biomass. MBC and MBN were positively correlated with C:Nimb but negatively correlated with C:Pimb and N:Pimb (Figure 4). These relationships suggest that microbial C and N accumulation were not only controlled by nutrient pool sizes but also by the balance between soil resource supply and microbial elemental composition [15,16]. The strong negative correlation between C:Nimb and N:Pimb further indicates that different dimensions of stoichiometric imbalance may interact with each other during wetland degradation.

### 4.4. Implications for Wetland Restoration and Land-Use Management

The results of this study indicate that microbial quotients and soil-microbial stoichiometric imbalance can serve as complementary indicators for evaluating soil ecological changes after wetland degradation. Traditional assessments often focus on soil C, N and P contents, microbial biomass or enzyme activities. However, these indicators cannot fully explain whether microorganisms are efficiently using soil resources or soil nutrient supply matches microbial demand [13,15]. By combining qMBC, qMBN and qMBP with C:Nimb, C:Pimb and N:Pimb, this study provides a more functional perspective on microbial adaptation under land-use conversion, and establishes a workable assessment framework for high-latitude, semi-arid wetlands.

The divergent responses of forestland and cropland also have important implications for land management. Forestland conversion increased N:Pimb, suggesting that nutrient balance, especially N-P matching, should be considered in restoration or afforestation practices. Cropland conversion increased qMBC, qMBN and qMBP, but also caused a strong increase in C:Nimb in 10CL. This indicates that long-term cropland use may enhance microbial activity while simultaneously increasing soil-microbial resource mismatch. Therefore, high microbial quotients in cropland should not be regarded as an entirely positive signal unless stoichiometric balance is also maintained.

For the A’er Xiang wetland, restoration strategies should not only focus on increasing soil nutrient contents, but also on maintaining the balance between soil nutrient supply and microbial demand. Measures that reduce soil disturbance, improve organic matter input, stabilize vegetation cover and regulate C, N and P inputs may help reduce soil-microbial stoichiometric imbalance. Recent studies in degraded wetlands have also emphasized the joint roles of plant biomass, soil properties, extracellular enzyme activities and soil nutrient availability in determining degradation and restoration trajectories [29,30,31].

The broader applicability of this framework across climatic regions remains an open question, because microbial responses to wetland degradation and land-use conversion are known to vary with environmental context. For instance, studies in restored wetlands in California have shown that microbial community composition is strongly mediated by site-specific hydrological management [32], while research on agricultural abandonment in Mediterranean peaty soils has revealed that post-agricultural soil chemical and biological recovery trajectories can differ markedly from those on sandy substrates [33]. In temperate post-agricultural ecosystems, land-use history, rather than restoration treatments alone, has been identified as the primary driver of soil-microbial biodiversity [34]. These comparative findings suggest that the diagnostic thresholds and reference conditions for microbial quotients and stoichiometric imbalance may need to be regionally calibrated, and that the framework developed here for a semi-arid sandy landscape would benefit from testing in wetlands under different climatic and edaphic settings. Overall, microbial quotients and stoichiometric imbalance provide useful indicators for evaluating the ecological consequences of wetland degradation and for guiding more targeted land-use management, and the present study offers a feasible approach for semi-arid sandy wetlands that can serve as a reference for similar regions.

## 5. Conclusions

This study investigated the effects of wetland degradation-induced land-use conversion on soil-microbial quotients and soil-microbial stoichiometric imbalance in the A’er Xiang lake marsh wetland. By comparing native wetland, forestland converted from wetland for five and ten years, and cropland converted from wetland for five and ten years, this study revealed distinct microbial resource-use patterns under different conversion pathways.

First, land-use conversion significantly altered soil-microbial quotients. Compared with native wetland, qMBC and qMBN decreased in the early stage of forestland conversion but increased with longer conversion duration, whereas cropland conversion generally showed higher qMBC, qMBN and qMBP values. Among the three microbial quotients indices, qMBP showed the strongest response, with all converted sites showing higher values than native wetland and 10CL showing the highest qMBP. This indicates that microbial P quotient is a sensitive indicator of wetland degradation-induced land-use conversion.

Second, forestland and cropland conversion showed different soil-microbial stoichiometric imbalance patterns. Long-term cropland conversion significantly increased C:Nimb, suggesting intensified mismatch between soil C:N supply and microbial C:N demand. In contrast, forestland conversion significantly increased N:Pimb, indicating that the forestland pathway mainly enhanced soil-microbial N:P imbalance. C:Pimb showed relatively large variations and did not differ significantly among the five treatment groups.

Third, microbial biomass was closely associated with soil-microbial stoichiometric imbalance. MBC and MBN were positively correlated with C:Nimb but negatively correlated with C:Pimb and N:Pimb, suggesting that microbial C and N accumulation were regulated not only by nutrient availability but also by the matching relationship between soil nutrient supply and microbial elemental demand.

Finally, correlation analysis showed that soil stoichiometry, microbial stoichiometry and extracellular enzyme stoichiometry were jointly linked with microbial quotients. These results confirm that microbial quotients and soil-microbial stoichiometric imbalance can serve as complementary indicators for evaluating microbial adaptation after wetland degradation. For the A’er Xiang lake marsh wetland, water availability is a key factor controlling wetland degradation and the primary constraint on future land-use conversion. The two conversion pathways serve distinct ecological functions in this context. Forestland conversion, given the proximity to the Horqin Sandy Land, primarily supports windbreak and sand fixation, making it a more suitable option for this sandy region. Cropland conversion, by contrast, requires sufficient water supply and active agricultural management to sustain soil fertility. Therefore, future land-use decisions in degraded lake marsh wetlands of sandy regions should consider both water availability and the specific ecological function of each conversion pathway. Microbial quotients and stoichiometric imbalance can provide complementary indicators for monitoring soil ecological responses during these conversions.

## Figures and Tables

**Table 1:** Study plots and main measured and calculated variables.

Land-Use/Conversion Type	Abbreviation	Original Land Type	Conversion Duration	Measured Variables	Calculated Variables
Native wetland	WL	Wetland	-	SOC, TN, TP; MBC, MBN, MBP; BG, NAG + LAP, PHOS	qMBC, qMBN, qMBP; C:Nimb, C:Pimb, N:Pimb
Wetland converted to forestland	5FL	Wetland	5 years		
Wetland converted to forestland	10FL	Wetland	10 years		
Wetland converted to cropland	5CL	Wetland	5 years		
Wetland converted to cropland	10CL	Wetland	10 years		

Note: WL = native wetland; 5FL and 10FL = wetland converted to forestland for 5 and 10 years; 5CL and 10CL = wetland converted to cropland for 5 and 10 years. SOC = soil organic carbon; TN = total nitrogen; TP = total phosphorus; MBC, MBN, MBP = microbial biomass carbon, nitrogen, phosphorus; BG = β-1,4-glucosidase; NAG + LAP = β-1,4-N-acetylglucosaminidase + L-leucine aminopeptidase; PHOS = acid phosphatase; qMBC, qMBN, qMBP = microbial quotients of C, N, P; C:Nimb, C:Pimb, N:Pimb = soil-microbial stoichiometric imbalance of C:N, C:P, N:P.

## Data Availability

The original contributions presented in this study are included in the article. Further inquiries can be directed to the corresponding author.
